# Why Is an Early Start of Training Related to Musical Skills in Adulthood? A Genetically Informative Study

**DOI:** 10.1177/0956797620959014

**Published:** 2020-12-14

**Authors:** Laura W. Wesseldijk, Miriam A. Mosing, Fredrik Ullén

**Affiliations:** 1 Department of Neuroscience, Karolinska Institutet; 2 Department of Psychiatry, Amsterdam UMC, University of Amsterdam; 3 Department of Medical Epidemiology and Biostatistics, Karolinska Institutet; 4 Melbourne School of Psychological Sciences, Faculty of Medicine, Dentistry, and Health Sciences, University of Melbourne

**Keywords:** sensitive period, musical training, musical expertise, twins, professional musicians

## Abstract

Experts in domains such as music or sports often start training early. It has been suggested that this may reflect a sensitive period in childhood for skill acquisition. However, it could be that familial factors (e.g., genetics) contribute to the association. Here, we examined the effect of age of onset of musical training on musical aptitude and achievement in professional musicians (*n* = 310) and twins (*n* = 7,786). In line with previous literature, results showed that an earlier age of onset was associated with higher aptitude and achievement in both samples. After we adjusted for lifetime practice hours, age of onset was associated only with aptitude (*p* < .001; achievement: *p* > .14). Twin analyses showed that the association with aptitude was fully explained by familial factors. Thus, these findings provide little support for a sensitive period for music but highlight that familiar factors play an important role for associations between age of onset of training and skills in adulthood.

There is a general belief that starting training early is essential to reach a high level of expertise in many fields, such as music or sports. If true, this might be because of a so-called sensitive period in early childhood. The concept of a sensitive period refers to a limited period of time in development during which effects of experience on the brain are unusually strong (Knudsen, 2004). In line with this concept, research has suggested that musical training during a sensitive period could result in the reshaping of neural circuitry with long-lasting benefits for performance later in life (Penhune, 2020; Watanabe, Savion-Lemieux, & Penhune, 2007). Most evidence for sensitive periods comes from research on language acquisition and visual and auditory functions, but a number of studies have explored effects of early versus late training in musical expertise. Schlaug, Jancke, Huang, Staiger, and Steinmetz (1995) reported that the anterior half of the corpus callosum was larger in musicians compared with nonmusicians but only in musicians who began musical training before the age of 7 years. Another study found that age of onset of musical training predicts absolute pitch (Baharloo, Johnston, Service, Gitschier, & Freimer, 1998). These studies, however, did not control for total amount of practice, which is likely to differ between early and late starters. Since then, several studies have compared groups of early- and late-starting musicians matched for total years of experience and still found associations between the age of onset of musical training and structural and functional properties of various auditory-motor brain structures (Bailey & Penhune, 2010, 2012; Bailey, Zatorre, & Penhune, 2014; Skoe & Kraus, 2013; Steele, Bailey, Zatorre, & Penhune, 2013; Vaquero et al., 2016), better performance on rhythmic synchronization tasks (Bailey & Penhune, 2013; Steele et al., 2013; Watanabe et al., 2007), and melody-discrimination skills (Ireland, Iyer, & Penhune, 2019).

Although these studies indicate that early training is of special importance for skill learning, there are also reasons to interpret the evidence for a sensitive period for music expertise with some caution. For example, the study by Ireland et al. (2019) found that children who received musical training before the age of 7 years outperformed later starting children on simple melody discrimination but not on transposed melody discrimination or complex rhythm synchronization. Furthermore, most studies matched early- and late-starting musicians on years of practice, which can result in an age difference between groups at follow up, with individuals starting early also being younger at the time of skill assessment, something that could potentially influence test outcomes because several cognitive domains are known to start declining in early adulthood (Salthouse, 2009). Controlling for accumulated practice and age may be a preferable way to correct for potential confounding of lifetime music practice. Additionally, it should be noted that some studies on musicians have revealed nonsignificant relations between age of onset of musical training and brain measures (Hutchinson, Lee, Gaab, & Schlaug, 2003; Keenan, Thangaraj, Halpern, & Schlaug, 2001). In sports, Güllich (2017) analyzed the practicing history of athletes. Interestingly, both medalists and nonmedalists started with organized general practice and competitions in various sports other than their later main sport at the same mean age (*M*s = 9.1 and 9.0 years, respectively). Notably, however, the medalists practiced other sports for longer periods and therefore specialized in their main sport significantly later than the nonmedalists. Another study found that earlier age of onset of training was not associated with success in senior international elite sport (Vaeyens, Güllich, Warr, & Philippaerts, 2009). In summary, although some research suggests that there may be a sensitive period during early childhood for skill learning in music and related domains, the studies have not always accounted for cumulative practice, and results are somewhat inconsistent.

Twin studies on musical expertise have found aptitude and achievement to be significantly influenced by genetic factors (male range = 38%–66%, female range = 20%–30%; Coon & Carey, 1989; Mosing, Madison, Pedersen, Kuja-Halkola, & Ullén, 2014; Vinkhuyzen, van der Sluis, Posthuma, & Boomsma, 2009; Wesseldijk, Mosing, & Ullén, 2019). This suggests that children with greater musical skills may be encouraged to seek out early musical training. In this scenario, genetic factors would contribute to the association between early training and expertise. On the other hand, musical training in early childhood may often be initiated by parents, which would imply a role for a shared familial influence (including genetics or shared family environment). Also, participation in extracurricular music education and cultural activities is correlated with socioeconomic status (Feldman & Matjasko, 2007; Kinney, 2008). Using twins, we can estimate genetic and environmental influences on age of onset of musical training, adjust for genetic and shared familial confounding when exploring the relationship between age of onset and musical expertise, and test whether such associations are in line with a causal hypothesis (McGue, Osler, & Christensen, 2010).

**Statement of Relevance**

A common observation is that successful musicians often start their musical training early. One much-discussed explanation for this is that there may be a sensitive period in childhood, during which the brain is particularly susceptible to musical stimulation. We show that the true story may be more complex than that. First, we found that one factor at play is that early-starting musicians simply tend to accumulate more training in total than those starting later. Secondly, we found that the relation between starting age and adult expertise is partly driven by common genetic factors, which influence both at which age someone starts training and musical expertise in adulthood. Therefore, an explanation could be that children, who for partly genetic reasons have high musical ability, also tend to be born into musically engaged families. These children show early signs of musicality, are encouraged to start practicing early, and grow up in a stimulating environment that benefits their musical expertise.

Here, we investigated whether an early onset of musical training has a causal impact on later musical expertise, in line with the sensitive-period hypothesis, and to what extent associations between age of onset of musical training and expertise can be explained by differences in accumulated practice and familial factors. We explored the influence of early training on two measures of expertise—musical aptitude and musical achievement—in a sample of 310 professional Swedish musicians and a population-based sample of 7,786 Swedish twins. There would be a significant association between age of onset of musical training and our two expertise measures in both samples, after adjustment for total amount of music practice and age, if there is a sensitive period for musical training. Furthermore, if the association between age of onset and expertise were causal, we expected the association to be independent of familial factors. Therefore, we also tested the association within identical twin pairs who share 100% of their genes as well as family environment.

## Method

### Participants

#### Musician sample

A sample of professional musicians was recruited via Swedish music institutions (orchestras, music schools) and through advertisements in various professional magazines for musicians. Participants were anonymous and logged onto the website for data collection using a personal login code distributed with the invitation letter. All participants gave informed consent. The study was approved by the regional ethical review board in Stockholm (Dnr 2013/1777-32). Data collection was conducted between November 2013 and March 2014, and a total of 570 individuals participated. Inclusion criteria for the present analyses were that (a) participants responded to an initial question about professional status by indicating either that they had finished their education as musicians and were professionally active or that they were still students in a college for music; (b) participants were between the ages of 27 and 54 years (i.e., the same age range as the twin sample); and (c) participants’ age of onset of musical training was between 2 and 18 years (as for the twin sample). This resulted in a final sample of 310 participants (173 women) with a mean age of 42.7 years (*SD* = 8.0) who were included in the analyses.

#### Twin sample

The Study of Twin Adults: Genes and Environment cohort includes approximately 32,000 adult twins born between 1959 and 1985 registered at the Swedish Twin Registry (Lichtenstein et al., 2002; Lichtenstein et al., 2006). In 2012 and 2013, data were collected in this cohort through an Internet survey on, among other things, musical achievement and musical aptitude. Of the 11,543 twin individuals who completed the survey, 7,786 reported having played music at some point in their life. Of these, 4,814 twins had also taken the Swedish Musical Discrimination Test, and 4,887 twins provided information on musical achievement. The participants were between the ages of 27 and 54 years (*M* = 40.7 years, *SD* = 7.8; for a more detailed description of the data collection, see Mosing et al., 2014). Collection and analyses of the twin data were approved by the regional ethical review board in Stockholm (Dnr 2011/570-31/5, Dnr 2012/1107/32).

### Measures

#### Age of onset of musical training

Participants were asked whether they had ever played a musical instrument or sang. Those who responded positively were asked at what age they started to play; whether they still played; and if not, at what age they stopped playing an instrument or singing. Individuals who reported a start age of 0 or 1 year were excluded from analyses (*n* = 25) because practicing music at those ages is unlikely and probably reflects rater bias. Also, we excluded participants who started musical training after 18 years of age (*n* = 183).

#### Hours of practice

Participants who had ever played an instrument or sang were asked to indicate the number of hours per week they practiced music (in 10 categories ranging from 0 hr, more than 6–9 hr, to more than 40 hr) during four age intervals (0–5 years, 6–11 years, 12–17 years, and 18 years until time of measurement). From these answers and information on the number of years they played music, we calculated estimates of total lifetime practice and practice until the age of 18 years.

#### Musical aptitude

Musical aptitude was measured using the Swedish Musical Discrimination Test (for details, see Ullén, Mosing, Holm, Eriksson, & Madison, 2014). This test is administered online and consists of three subtests: pitch, melody, and rhythm discrimination. Overall musical aptitude was calculated as the mean of standardized scores on these three subscales. The musical-aptitude score was normally distributed without outliers. See Ullén et al. (2014) for a more detailed description of the different tests and psychometric validation of the Swedish Musical Discrimination Test.

#### Musical achievement in the musician sample

Musical achievement in the professional musicians was estimated using a novel instrument, the Swedish Musical Achievement Questionnaire. The questionnaire was designed to tap into differences in achievement within a professional sample and consists of 23 items with seven or eight ordinal response options. Questions ask about the number of composed and performed works; pupils and students; CD, radio, and television recordings; national and international reviews; awards; participation on competition juries; and success of pupils. An English translation of the full questionnaire is available on request from the corresponding author. A sum score of all 23 items was used as a measure of overall musical achievement.

#### Musical achievement in the twin sample

Musical achievement in the twin sample was measured with an adapted and translated version of the Creative Achievement Questionnaire, previously used in Swedish population studies (Carson, Peterson, & Higgins, 2005; Mosing et al., 2015; Mosing, Verweij, Abe, de Manzano, & Ullén, 2016; Wesseldijk et al., 2019). Achievements in several art and science domains, including music, are measured using a 7-point scale. The ratings for music range from 1 (*I am not engaged in music at all*) via 4 (*I have played or sung, or my music has been played in public concerts in my home town, but I have not been paid for this*) to 7 (*I am professionally active as a musician and have been reviewed/featured in national or international media and/or have received an award for my musical activities*).

### Statistical analyses

All measures included in the analyses, except for sex, were standardized. Hierarchical linear regression analyses were performed in STATA (StataCorp, 2015) in both samples to explore the effect of age of onset of musical training on musical aptitude and on musical achievement. First, sex, age, and age of onset were included in the analyses, and total practice was added later. Additionally, we performed three linear regression analyses to estimate the effect of age of onset on the three subtests of the musical-aptitude test (i.e., rhythm-, melody-, and pitch-discrimination scores). In a sensitivity analysis, we repeated all regression analyses in both samples, predicting musical aptitude and achievement with and without controlling for total practice by age of onset as a binary independent variable, with 0 indicating starting musical training before the age of 8 years and 1 indicating starting musical training at or after the age of 8 years. To correct for relatedness in the twin sample, we used the robust standard error estimator for clustered observations.

Cotwin control analyses in identical twins were conducted to explore whether the significant association between age of onset of musical training (both continuous and binary) and musical aptitude remained after controlling for genetic and shared environmental factors. Because monozygotic twins are genetically identical and share their rearing family environment, exploring within-pair effects in identical twins allows for estimation of the effect free from familial confounding (McGue et al., 2010). If the association between age of onset of musical training and expertise observed in the main analyses (population level) were entirely free from familial confounding, hence reflecting a “true” causal effect, we would expect the within-twin association to be of similar magnitude. However, if the association were partly or wholly due to familial confounding, we would expect the association to diminish or become zero. Within-pair linear regression analyses were conducted using the “xtreg fe” statement in STATA to stratify by twin pair. Only complete identical twin pairs discordant in age of onset of musical training—299 monozygotic pairs in the present sample—contributed to the within-pair analyses. The analyses were repeated with hours of practice included as a covariate. Correcting for sex and age was not necessary because each monozygotic twin was matched to his or her cotwin.

Classical twin modeling was performed using structural equation modeling in OpenMx in the R programming environment (Boker et al., 2011). With the use of twin data, we partitioned the variance in age of onset of musical training into additive genetic (*A*), shared environmental (*C*), and nonshared environmental (*E*) components. Because monozygotic twins share approximately 100% of their genes, and dizygotic twins share 50% on average, higher monozygotic than dizygotic twin correlations indicate that genetic factors play a role for age of onset of musical training. If the dizygotic correlations are more than half the monozygotic twin correlations, it implies that shared environmental influences are of importance for age of onset. The remaining part of the variance is attributed to nonshared environmental influences and includes measurement error. In a similar way, *A, C*, and *E* influences can be estimated on the covariance between two traits on the basis of the cross-twin/cross-trait correlations. An influence of *E* factors on the covariance between age of onset of musical training and musical aptitude or achievement is expected in case of a causal association, whereas influences of *A* or *C* on the covariance indicate that familial factors are of importance. We first fitted three univariate *ACE* models to estimate genetic and environmental effects on variation in age of onset of musical training, musical aptitude, and musical achievement. Second, we fitted two bivariate *ACE* models to estimate genetic and environmental influences on the covariance between age of onset and (a) musical aptitude and (b) musical achievement.

Before fitting the genetic models to the twin data, we fitted a saturated model with means and correlations per zygosity and sex, in which we tested for sex differences in age of onset of musical training with the use of the likelihood-ratio test. The negative log likelihood (−2LL) of a submodel was subtracted from the −2LL of the more general saturated model. Using the difference in log likelihood and the difference in the number of degrees of freedom between the models, a chi-square test showed whether constraints significantly deteriorated the fit (α < .01). First, we constrained the means to be equal across sex to test for sex differences in mean age of onset. Second, we constrained the monozygotic and dizygotic twin correlations to be equal across the sexes to test for sex differences in the contributions of *A, C*, and *E* on age of onset (quantitative sex differences). Third, we constrained the correlation for same-sex dizygotic twins to be equal to the correlation in opposite-sex dizygotic twins to test whether different genes or different shared environmental factors operate in influencing age of onset in male and female participants (qualitative sex differences).

## Results

### Descriptive statistics

Female participants started musical training, on average, at an earlier age than male participants, and participants from the musician sample started earlier than participants in the twin sample (see Table 1). The proportion of correct responses on the musical-aptitude subtests of pitch, melody, and rhythm discrimination are also shown in Table 1, confirming higher levels of musical aptitude in the professional musician sample compared with the population-based twin sample. Correlations between musical aptitude, musical achievement, age of onset of musical training, age, and hours of practice are reported in Table 2.

**Table 1.** Descriptive Statistics for the Two Samples

Variable	Professional musicians	Twins
Mean age of onset of musical training (years)		
Women	6.68 (1.85)	8.46 (2.09)
Men	8.06 (2.73)	9.35 (2.56)
Median age of onset of musical training (years)		
Women	7	9
Men	8	8
Proportion of correct responses on musical-aptitude subtests		
Pitch discrimination	.91 (.08)	.67 (.18)
Melody discrimination	.64 (.16)	.37 (.16)
Rhythm discrimination	.97 (.05)	.85 (.12)

Note: Values in parentheses are standard deviations.

**Table 2.** Correlations Among Musical Aptitude, Musical Achievement, Age of Onset of Musical Training, Age, and Total Practice for the Musician and Twin Samples

Sample and variable	Musical aptitude	Musical achievement	Age of onset	Age
Musician sample				
Musical achievement	.08			
Age of onset	−.18	−.11		
Age	.04	.28	.04	
Total practice	.05	.44	−.12	.79
Twin sample				
Musical achievement	.40			
Age of onset	−.12	−.17		
Age	−.08	−.04	−.01	
Total practice	.30	.61	−.26	.19

### Regression analyses

**Fig. 1.**
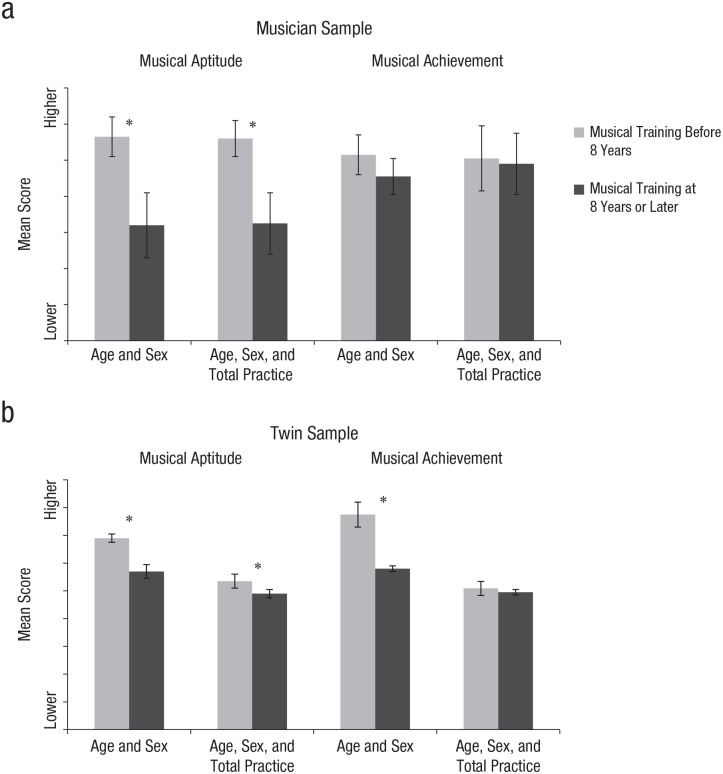
Mean residual score for musical aptitude and musical achievement in the musician sample (a) and twin sample (b) adjusted for age and sex and for age, sex, and total practice. For each analysis, results are shown separately for individuals who started musical training before the age of 8 years and at 8 years or later. Asterisks indicate significant differences between age groups (*p* < .01). Error bars indicate confidence intervals.

In both the musician sample and twin sample, regression analyses showed that age of onset of musical training significantly predicted musical aptitude and musical achievement (see Table 3, Model 1). When we added total practice to the analyses, the association with musical aptitude remained, but the association with musical achievement became nonsignificant (see Table 3, Model 2). In other words, a younger age at onset of musical training predicts higher levels of musical aptitude but does not influence musical achievement once total practice is held constant. Further analyses showed that in both samples, earlier age of onset of musical training significantly predicted higher levels of pitch discrimination (β = −0.07, *p* < .001 in musicians; β = −0.30, *p* < .001 in twins) but not rhythm discrimination (β = −0.03, *p* = .69 in musicians; β = −0.02, *p* = .09 in twins), when controlling for total practice. Only in the twin sample did a lower age of onset predict higher levels of melody discrimination (β = −0.09, *p* = .23 in musicians; β = −0.05, *p* = .001 in twins). Results were similar when age of onset of musical training was included as a binary variable, testing for age windows of below and at or above 8 years, with the exception that in the musician sample, age of onset was unrelated to musical achievement, even without total practice in the model (see Fig. 1; for the betas, see Table S1 in the Supplemental Material available online).

**Table 3.** Coefficients From the Hierarchical Regression Analyses on the Effect of Age of Onset of Musical Training on Musical Aptitude and Musical Achievement

Variable	Sex	Age	Age of onset	Total practice
Musician sample				
Musical aptitude				
Model 1	0.26 (*p* = .07)	0.05 (*p* = .47)	**−0.21** (*p* = .005)	
Model 2	0.27 (*p* = .07)	0.08 (*p* = .51)	**−0.21** (*p* = .006)	−0.03 (*p* = .78)
Musical achievement				
Model 1	**0.74** (*p* < .001)	**0.30** (*p* < .001)	**−0.16** (*p* = .003)	
Model 2	**0.70** (*p* < .001)	−0.13 (*p* = .12)	−0.08 (*p* = .14)	**0.55** (*p* < .001)
Twin sample				
Musical aptitude				
Model 1	**0.25** (*p* < .001)	**−0.06** (*p* < .001)	**−0.12** (*p* < .001)	
Model 2	**0.20** (*p* < .001)	**−0.10** (*p* < .001)	**−0.05** (*p* < .001)	**0.22** (*p* < .001)
Musical achievement				
Model 1	**0.18** (*p* < .001)	**−0.05** (*p* < .001)	**−0.20** (*p* < .001)	
Model 2	0.03 (*p* = .33)	**−0.17** (*p* < .001)	−0.01 (*p* = .59)	**0.65** (*p* < .001)

Note: Model 1 included sex, age, and age of onset of musical training, whereas Model 2 included sex, age, total practice, and age of onset of musical training. All variables (except sex) were standardized. Values in boldface are significant.

We tested for collinearity, and the tolerance values, ranging from .88 (hours of practice) to .96 (age), as well as the variance-inflation factors, which were all below 1.13, indicated no violation. To make sure that we did not overadjust, we repeated the analyses, adjusting for total practice until age 18 years only as a sensitivity analysis. All results remained the same.

### Cotwin control analyses

The mean discordance in years for starting musical training was 1.48 years (*SD* = 1.68) for monozygotic twins, 1.80 years (*SD* = 1.81) for dizygotic twins, and 2.01 years (*SD* = 2.10) for opposite-sex twins. Within-pair linear regressions in identical twin pairs showed no significant effect of age of onset of musical training on musical aptitude (β = 0.00, *p* = .93). When we added hours of practice to the analyses, the results did not change (β = 0.01, *p* = .87). Similarly, within-pair analyses in identical pairs showed no significant effect of age of onset before the age of 8 years versus age of onset at age 8 years or later on musical aptitude (β = 0.02, *p* = .66; β = 0.03, *p* = .60 when analyses controlled for total practice). In addition, age of onset did not predict any of the outcomes on the subtests of musical aptitude within identical twin pairs when analyses controlled for total practice (rhythm discrimination: β = −0.03, *p* = .56; melody discrimination: β = −0.02, *p* = .71; pitch discrimination: β = 0.08, *p* = .13).

### Classical twin modeling

Within-pair correlations for age of onset of musical training and cross-twin/cross-trait correlations between age of onset of musical training and musical aptitude and between age of onset and musical achievement are presented in Table 4. We did not detect quantitative sex differences—that is, same-sex male and same-sex female twin pairs could be constrained to be equal, χ^2^(2) = 8.66, *p* = .01—or qualitative sex differences—that is, correlations between same-sex and opposite-sex dizygotic twins could be constrained to be equal, χ^2^(1) = 4.39, *p* = .04.

**Table 4.** Within-Pair Correlations for Age of Onset of Musical Training and Cross-Twin/Cross-Trait Correlations for Age of Onset of Musical Training and Musical Achievement

Correlation and group	*r*
Age of onset within pairs	
Monozygotic male	.39 [.28, .48]
Monozygotic female	.55 [.48, .61]
Dizygotic male	.34 [.21, .46]
Dizygotic female	.35 [.24, .45]
Dizygotic opposite sex	.21 [.10, .32]
Total monozygotic	.49 [.43, .54]
Total dizygotic	.29 [.22, .35]
Age of onset and musical aptitude across twins	
Total monozygotic	.14 [.09, .20]
Total dizygotic	.13 [.08, .19]
Age of onset and musical achievement across twins	
Total monozygotic	.20 [.15, .25]
Total dizygotic	.17 [.12, .22]

Note: Values in brackets are 95% confidence intervals.

On the basis of these outcomes, we fitted an *ACE* model to the twin data, allowing the means to differ between male and female participants, to estimate genetic and environmental effects on variation in age of onset of musical training. Genetic factors (39%, 95% CI = [23, 54]), shared environmental factors (10%, 95% CI = [0, 23]), and unique environmental factors (51%, 95% CI = [45, 56]) contributed to individual differences in age of onset of musical training (see Table 5). Furthermore, the two bivariate *ACE* models showed that the association between age of onset of musical training and musical aptitude as well as the association between age of onset and musical achievement were both fully explained by familial factors, namely, genetic and shared environmental influences. There was no significant influence of nonshared environmental factors (see Table 5, bivariate heritability).

**Table 5.** Standardized Estimates of Additive Genetic (*A*), Shared Environmental (*C*), and Nonshared Environmental (*E*) Influences on Age of Onset of Musical Training, Musical Aptitude, and Musical Achievement and the Associations Between Age of Onset and Musical Aptitude and Age of Onset and Musical Achievement Explained by *A, C*, and *E* Influences

Factor	Heritability			Bivariate heritability	
	Age of onset	Musical aptitude	Musical achievement	Age of onset and musical aptitude	Age of onset and musical achievement
*A*	39%	66%	36%	49%	16%
*C*	10%	7%	24%	51%	84%
*E*	51%	28%	40%	0%	0%

Note: The *ACE* estimates for musical aptitude and achievement shown here were collapsed across sex, whereas women and men were analyzed separately in previous studies using this sample (for musical aptitude, see Mosing, Madison, Pedersen, Kuja-Halkola, & Ullén, 2014; for musical achievement, see Wesseldijk, Mosing, & Ullén, 2019).

## Discussion

Here, we examined whether musical training at a younger age leads to higher levels of musical expertise when controlling for the effects of total practice and familial factors, as would be predicted from the hypothesis that there is a sensitive period for musical training in childhood. In both professional musicians and twins, an earlier age of onset of musical training was associated with higher aptitude and achievement. However, when we controlled for lifetime practice, associations between age of onset and achievement became insignificant, whereas age of onset still predicted aptitude. The latter association disappeared, in turn, when we controlled for familial liability in a cotwin control design. Further twin analyses showed that the associations between age of onset of musical training and musical aptitude and between age of onset and musical achievement were fully explained by familial factors (i.e., shared genetic and shared environmental factors), in line with our cotwin control findings.

In both samples, an earlier age of onset of musical training was associated with higher musical aptitude and musical achievement, but when analyses controlled for lifetime practice, age of onset significantly predicted only higher levels of musical aptitude. Whereas this highlights the importance of adjusting for lifetime practice when exploring the above associations, it also lends further support to the findings of associations between age of onset of musical training and performance on some musical tasks reported in earlier studies (Bailey & Penhune, 2010, 2012, 2013; Bailey et al., 2014; Ireland et al., 2019; Skoe & Kraus, 2013; Steele et al., 2013; Vaquero et al., 2016; Watanabe et al., 2007). The consistency across our two samples, a professional-musician and population-based twin sample, strengthens these findings and suggests a similar effect of age of onset of musical training in a wide range of musical expertise. More importantly, our findings suggest a mediating effect of total practice on the relationship between age of onset of musical training and musical achievement but not on musical aptitude. Importantly, cumulative lifetime practice has been shown to be influenced by genetic factors (Mosing et al., 2014) and therefore does not reflect only unique environmental influences. Further, in both samples, age of onset of musical training significantly predicted higher levels of pitch discrimination but not rhythm discrimination. Only in the population-based sample did an earlier age of onset predict higher levels of melody discrimination. This is in line with the finding by Ireland and colleagues (2019) that children who received musical training before the age of 7 years outperformed children who started later on simple melody discrimination but not complex rhythm synchronization. Last, when we treated age of onset as a binary variable to test for an age window of below and at or above 8 years old, our findings remained the same.

The twin sample allowed us to extend our analyses to control for familial confounding, thereby further investigating causality, as well as to estimate the influence of genetic, shared environmental, and nonshared environmental factors on age of onset of musical training and its relationship with expertise. The twin analyses provided no evidence for a causal effect of early training in such associations. First, we found the association between age of onset of musical training (continuous or binary) and musical aptitude or achievement to diminish (close to zero) when controlling for familial liability. Further, the associations were fully explained by familial factors (i.e., common genetic and shared environmental). Because unique environmental factors did not play a role in the association between age of onset of musical training and musical expertise, the data provide little support for a causal association. We wish to emphasize that these findings do not necessarily rule out the existence of a sensitive period. Importantly, however, our findings provide clear evidence for the importance of shared familiar factors in associations between age of onset and adult performance.

As mentioned before, genetic predispositions may make some children more likely to start musical training early. They may be encouraged by other people who recognize their talent and may to a higher degree seek out, show interest in, and have access to a musical environment. More musical parents may not only pass their genetic predisposition to their children but also provide both access to early musical training and a musically enriched childhood environment that enhances musical expertise. This is an example of gene–environment correlation, in which genetics and shared environmental factors may influence the association between age of onset of musical training and later expertise. For future research, children-of-twins and adoption studies are genetically informative designs that offer possibilities to further explore gene–environment correlation.

There are some limitations of this study. First, age of onset of musical training was self-reported, which may introduce a rater or recall bias. Another possibility is that parents who are aware that their children are monozygotic twins treat them more similarly compared with parents of dizygotic twins with regard to early musical training. This would mean a violation of the equal-environment assumption (i.e., that, on average, both monozygotic and dizygotic twins are treated equally similarly), also causing an upward bias in the heritability estimates. Although we were not able to control for this in our study, multiple earlier studies have shown that the assumption generally holds (Derks, Dolan, & Boomsma, 2006). The absence of an effect of age of onset of musical training on rhythm discrimination in professional musicians should be interpreted with caution because strong ceiling effects were found in the musician sample on this subtest. Last, we note that the mean age of onset is significantly higher in male than in female participants. However, additional regression analyses separately for sex did not change the findings. One major strength of this study is the availability of both a professional musician sample and a large population-based twin sample. This allowed us to fully explore the association between age of onset of musical training and musical expertise while controlling for confounds between genetic and shared environmental factors.

The present study is, to our knowledge, the largest and only genetically informative study to focus on whether starting musical training at a younger age leads to higher levels of musical expertise. When controlling for lifetime practice, we found that an earlier age of onset of musical training predicted higher levels of musical aptitude in adulthood in professional musicians and in the general population. However, the association diminished when analyses controlled for familial liability in a cotwin control design. This, together with the finding that the association between age of onset of musical training and musical aptitude was fully explained by familial factors, suggests that a genetic predisposition for music may make children start musical training at a younger age. Thus, our findings provide little direct support that early training has a specific, causal effect on later performance and achievement; rather, they highlight the importance of taking into account cumulative measures of practice as well as genetic and shared environmental factors when studying sensitive periods and effects of an early age of onset of musical training on expertise in later life.

## Supplemental Material

sj-docx-1-pss-10.1177_0956797620959014 – Supplemental material for Why Is an Early Start of Training Related to Musical Skills in Adulthood? A Genetically Informative Study Click here for additional data file. Supplemental material, sj-docx-1-pss-10.1177_0956797620959014 for Why Is an Early Start of Training Related to Musical Skills in Adulthood? A Genetically Informative Study by Laura W. Wesseldijk, Miriam A. Mosing and Fredrik Ullén in Psychological Science

## Transparency

*Action Editor:* Leah Somerville

*Editor:* Patricia J. Bauer

*Author Contributions*

M. A. Mosing and F. Ullén share senior authorship. All the authors designed the research. L. W. Wesseldijk and M. A. Mosing performed the research and analyzed the data. L. W. Wesseldijk and M. A. Mosing wrote the manuscript, and F. Ullén provided critical revisions. All the authors approved the final manuscript for submission.

**Open Practices:** The data sets generated and analyzed in the current study are not publicly available because registry data were used, but the data sets are available from the Swedish Twin Registry on request. Details on the application procedures for data usage are available on the home page of the registry (http://ki.se/en/research/swedish-twin-registry-for-researchers). The design and analysis plans for this study were not preregistered.

## References

[bibr1-0956797620959014] Baharloo S. Johnston P. A. Service S. K. Gitschier J. Freimer N. B. (1998). Absolute pitch: An approach for identification of genetic and nongenetic components. The American Journal of Human Genetics, 62, 224–231. doi:10.1086/301704 9463312 PMC1376881

[bibr2-0956797620959014] Bailey J. A. Penhune V. B. (2010). Rhythm synchronization performance and auditory working memory in early- and late-trained musicians. Experimental Brain Research, 204(1), 91–101. doi:10.1007/s00221-010-2299-y 20508918

[bibr3-0956797620959014] Bailey J. A. Penhune V. B. (2012). A sensitive period for musical training: Contributions of age of onset and cognitive abilities. Annals of the New York Academy of Sciences, 1252, 163–170. doi:10.1111/j.1749-6632.2011.06434.x 22524355

[bibr4-0956797620959014] Bailey J. A. Penhune V. B. (2013). The relationship between the age of onset of musical training and rhythm synchronization performance: Validation of sensitive period effects. Frontiers in Neuroscience, 7, Article 227. doi:10.3389/fnins.2013.00227 PMC3843222 24348323

[bibr5-0956797620959014] Bailey J. A. Zatorre R. J. Penhune V. B. (2014). Early musical training is linked to gray matter structure in the ventral premotor cortex and auditory-motor rhythm synchronization performance. Journal of Cognitive Neuroscience, 26, 755–767. doi:10.1162/jocn_a_00527 24236696

[bibr6-0956797620959014] Boker S. Neale M. Maes H. Wilde M. Spiegel M. Brick T. Fox J. (2011). OpenMx: An open source extended structural equation modeling framework. Psychometrika, 76, 306–317. doi:10.1007/s11336-010-9200-6 23258944 PMC3525063

[bibr7-0956797620959014] Carson S. H. Peterson J. B. Higgins D. M. (2005). Reliability, validity, and factor structure of the Creative Achievement Questionnaire. Creativity Research Journal, 17(1), 37–50.

[bibr8-0956797620959014] Coon H. Carey G. (1989). Genetic and environmental determinants of musical ability in twins. Behavioral Genetics, 19, 183–193. 10.1007/BF01065903 2719622

[bibr9-0956797620959014] Derks E. M. Dolan C. V. Boomsma D. I. (2006). A test of the equal environment assumption (EEA) in multivariate twin studies. Twin Research and Human Genetics, 9, 403–411. doi:10.1375/183242706777591290 16790150

[bibr10-0956797620959014] Feldman A. F. Matjasko J. L. (2007). Profiles and portfolios of adolescent school-based extracurricular activity participation. Journal of Adolescence, 30, 313–332. doi:10.1016/j.adolescence.2006.03.004 16678248

[bibr11-0956797620959014] Güllich A. (2017). International medallists’ and non-medallists’ developmental sport activities – a matched-pairs analysis. Journal of Sports Sciences, 35, 2281–2288. doi:10.1080/02640414.2016.1265662 27923322

[bibr12-0956797620959014] Hutchinson S. Lee L. H. Gaab N. Schlaug G. (2003). Cerebellar volume of musicians. Cerebral Cortex, 13, 943–949. 12902393 10.1093/cercor/13.9.943

[bibr13-0956797620959014] Ireland K. Iyer T. A. Penhune V. B. (2019). Contributions of age of start, cognitive abilities and practice to musical task performance in childhood. PLOS ONE, 14(4), Article e0216119. doi:10.1371/journal.pone.0216119 PMC6483258 31022272

[bibr14-0956797620959014] Keenan J. P. Thangaraj V. Halpern A. R. Schlaug G. (2001). Absolute pitch and planum temporale. NeuroImage, 14, 1402–1408. doi:10.1006/nimg.2001.0925 11707095

[bibr15-0956797620959014] Kinney D. W. (2008). Selected demographic variables, school music participation, and achievement test scores of urban middle school students. Journal of Research in Music Education, 56, 145–161. doi:10.1177/0022429408322530

[bibr16-0956797620959014] Knudsen E. I. (2004). Sensitive periods in the development of the brain and behavior. Journal of Cognitive Neuroscience, 16, 1412–1425. doi:10.1162/0898929042304796 15509387

[bibr17-0956797620959014] Lichtenstein P. De Faire U. Floderus B. Svartengren M. Svedberg P. Pedersen N. L. (2002). The Swedish Twin Registry: A unique resource for clinical, epidemiological and genetic studies. Journal of Internal Medicine, 252, 184–205. 12270000 10.1046/j.1365-2796.2002.01032.x

[bibr18-0956797620959014] Lichtenstein P. Sullivan P. F. Cnattingius S. Gatz M. Johansson S. Carlström E.. . . Pedersen N. L. (2006). The Swedish Twin Registry in the third millennium: An update. Twin Research and Human Genetics, 9, 875–882. doi:10.1375/183242706779462444 17254424

[bibr19-0956797620959014] McGue M. Osler M. Christensen K. (2010). Causal inference and observational research: The utility of twins. Perspectives on Psychological Science, 5, 546–556. doi:10.1177/1745691610383511 21593989 PMC3094752

[bibr20-0956797620959014] Mosing M. A. Madison G. Pedersen N. L. Kuja-Halkola R. Ullén F. (2014). Practice does not make perfect: No causal effect of music practice on music ability. Psychological Science, 25, 1795–1803. doi:10.1177/0956797614541990 25079217

[bibr21-0956797620959014] Mosing M. A. Verweij K. J. H. Abe C. de Manzano O. Ullén F. (2016). On the relationship between domain-specific creative achievement and sexual orientation in Swedish twins. Archives of Sexual Behavior, 45, 1799–1806. doi:10.1007/s10508-016-0708-4 26969321

[bibr22-0956797620959014] Mosing M. A. Verweij K. J. H. Madison G. Pedersen N. L. Zietsch B. P. Ullén F. (2015). Did sexual selection shape human music? Testing predictions from the sexual selection hypothesis of music evolution using a large genetically informative sample of over 10,000 twins. Evolution and Human Behavior, 36, 359–366.

[bibr23-0956797620959014] Penhune V. B. (2020). A gene-maturation-environment model for understanding sensitive period effects in musical training. Current Opinion in Behavioral Sciences, 36, 13–22. 10.1007/7854_2021_250 34435343

[bibr24-0956797620959014] Salthouse T. A. (2009). When does age-related cognitive decline begin? Neurobiology of Aging, 30, 507–514. doi:10.1016/j.neurobiolaging.2008.09.023 19231028 PMC2683339

[bibr25-0956797620959014] Schlaug G. Jancke L. Huang Y. Staiger J. F. Steinmetz H. (1995). Increased corpus callosum size in musicians. Neuropsychologia, 33, 1047–1055. 8524453 10.1016/0028-3932(95)00045-5

[bibr26-0956797620959014] Skoe E. Kraus N. (2013). Musical training heightens auditory brainstem function during sensitive periods in development. Frontiers in Psychology, 4, Article 622. doi:10.3389/fpsyg.2013.00622 PMC3777166 24065935

[bibr27-0956797620959014] StataCorp. (2015). Stata statistical software: Version 14 [Computer software]. College Station, TX: Author.

[bibr28-0956797620959014] Steele C. J. Bailey J. A. Zatorre R. J. Penhune V. B. (2013). Early musical training and white-matter plasticity in the corpus callosum: Evidence for a sensitive period. The Journal of Neuroscience, 33, 1282–1290. doi:10.1523/JNEUROSCI.3578-12.2013 23325263 PMC6704889

[bibr29-0956797620959014] Ullén F. Mosing M. A. Holm L. Eriksson H. Madison G. (2014). Psychometric properties and heritability of a new online test for musicality, the Swedish Musical Discrimination Test. Personality and Individual Differences, 63, 87–93. doi:10.1016/j.paid.2014.01.057

[bibr30-0956797620959014] Vaeyens R. Güllich A. Warr C. R. Philippaerts R. (2009). Talent identification and promotion programmes of Olympic athletes. Journal of Sports Sciences, 27, 1367–1380. doi:10.1080/02640410903110974 19787538

[bibr31-0956797620959014] Vaquero L. Hartmann K. Ripollés P. Rojo N. Sierpowska J. Francois C.. . . Altenmüller E. (2016). Structural neuroplasticity in expert pianists depends on the age of musical training onset. NeuroImage, 126, 106–119. doi:10.1016/j.neuroimage.2015.11.008 26584868

[bibr32-0956797620959014] Vinkhuyzen A. A. van der Sluis S. Posthuma D. Boomsma D. I. (2009). The heritability of aptitude and exceptional talent across different domains in adolescents and young adults. Behavioral Genetics, 39, 380–392. doi:10.1007/s10519-009-9260-5 PMC2688647 19288254

[bibr33-0956797620959014] Watanabe D. Savion-Lemieux T. Penhune V. B. (2007). The effect of early musical training on adult motor performance: Evidence for a sensitive period in motor learning. Experimental Brain Research, 176, 332–340. doi:10.1007/s00221-006-0619-z 16896980

[bibr34-0956797620959014] Wesseldijk L. W. Mosing M. A. Ullén F. (2019). Gene–environment interaction in expertise: The importance of childhood environment for musical achievement. Developmental Psychology, 55, 1473–1479. doi:10.1037/dev0000726 30883154

