# Supplementary material for: Why Is an Early Start of Training Related to Musical Skills in Adulthood? A Genetically Informative Study
Source: Psychol Sci. 2020 Dec 14;32(1):3–13. doi: 10.1177/0956797620959014 (PMC7809336; doi:10.1177/0956797620959014)
Supplement: sj-docx-1-pss-10.1177_0956797620959014 – Supplemental material for Why Is an Early Start of Training Related to Musical Skills in Adulthood? A Genetically Informative Study [file sj-docx-1-pss-10.1177_0956797620959014.docx]

**Supplementary Online Materials - Reviewed**

Supplementary Table 1. Standardized regression coefficients (β) and p-values from the hierarchical regression analyses in the musician and twin sample on the effect of AoO before the age of 8 or at age 8 or later (0 is before, 1 is after) on musical aptitude and musical achievement. Model 1 included sex, age and AoO, while Model 2 included sex, age, total hours of practice and AoO. All variables (except sex) were standardized.

|  | **Sex** | **Age** | **AoO before 8 or after** | | **Total practice** |
| --- | --- | --- | --- | --- | --- |
| *Musician sample* | | | *N=228 before 8, N=82 after 8* | |  |
| Musical aptitude | | |  |  | |
| Model 1 | 0.25  (*p* = 0.08) | 0.04  (*p* = 0.56) | -0.50*  (*p* < 0.001) | - | |
| Model 2 | 0.25  (*p* = 0.09) | 0.03  (*p* = 0.79) | -0.50*  (*p* = 0.003) | -0.01  (*p* = 0.90) | |
| Musical achievement | | | | | |
| Model 1 | 0.68*  (*p* < 0.001) | 0.29*  (*p* < 0.001) | -0.12  (p = 0.31) | - | |
| Model 2 | 0.66*  (*p* < 0.001) | -0.15  (*p* = 0.06) | -0.03  (*p* = 0.81) | 0.58*  (*p* < 0.001) | |
| *Twin sample* | | | *N=1,348 before 8, N=3,321 after 8* |  | |
| Musical aptitude | | |  |  | |
| Model 1 | 0.22*  (*p* < 0.001) | -0.06*  (*p* < 0.001) | -0.20*  (*p* < 0.001) | - | |
| Model 2 | 0.19*  (*p* < 0.001) | -0.10*  (*p* < 0.001) | -0.06*  (*p* < 0.01) | 0.22*  (*p* < 0.001) | |
| Musical achievement | | | | | |
| Model 1 | 0.14*  (*p* < 0.001) | -0.05*  (*p* < 0.01) | -0.41*  (*p* < 0.001) | - | |
| Model 2 | 0.03  (*p* = 0.34) | -0.17*  (*p* < 0.001) | -0.03  (*p* = 0.35) | 0.65*  (*p* < 0.001) | |
